# Inverting the lady’s slipper orchid: development and active–passive mechanisms in a ‘living machine’

**DOI:** 10.1093/jxb/erae444

**Published:** 2025-01-08

**Authors:** Nick Rowe

**Affiliations:** AMAP, Univ. Montpellier, CNRS, CIRAD, INRA, IRD, Montpellier, France

**Keywords:** Bio-inspired technologies, biomechanics, development, functional traits

## Abstract

This article comments on:

**Cardoso JCF, Johnson SD, Oliveira PE. 2025.** Flowers meet Newton: testing the role of gravitational pull in resupination of orchid flowers. Journal of Experimental Botany **76**, 433–444. https://doi.org/10.1093/jxb/erae413

This article comments on:

**Cardoso JCF, Johnson SD, Oliveira PE. 2025.** Flowers meet Newton: testing the role of gravitational pull in resupination of orchid flowers. Journal of Experimental Botany **76**, 433–444. https://doi.org/10.1093/jxb/erae413

**Resupination, the downwards re-orientation of flowers, is a key trait for angiosperms, especially orchids. The study of a lady’s slipper orchid from Brazil by Cardoso *et al.* (2025) shows that the seemingly basic mechanism is far from simple and that both passive and active mechanisms are at play. Turning upside down is not only a process of gravitropism; it also requires deft positioning of the flower mass to act as a counterweight, and compliance of the flower stalk to act as a hinge. Developmental timing, functional traits, and viewing functional ensembles as living machines broaden our understanding of evolutionary process and drive novel bio-inspired technologies.**

## Biomechanics and active–passive processes in plants

A mechanical component probably exists in most functional traits of animals and plants. It is undoubtedly true for plant movements—stem, root, leaf, and flower orientation, where the outward movements are impressive, but the underlying mechanisms are complex and not very visible. Movement by growth towards light or against gravity requires active growth processes: meristematic growth, cell division, cell expansion, tissue differentiation, wall thickening, and lignification. Leaves, stems, and flowers also depend on pre-formed mechanical organization to optimize support, stability, and safety against failure at any given moment. Such mechanisms can include fine-tuned geometric placement of physiologically costly mechanical tissues as well as stiffness or compliance to adaptively resist or deflect under bending and torsion moments. In contrast to active growth processes, these kinds of structural traits function in a more passive way once formed. For example, the deflection and re-configuration of leaves and shoots in strong winds which reduce the overall sail area and mechanical stress of a tree depend on pre-formed structural and mechanical innovations that deploy passive reactions to external stresses (Ennos, 1993; Vogel, 1992). There is little or no active growth and development during these reconfiguration processes, so they are entirely passive but nevertheless functional and adaptive. In other situations involving complex plant movements, the distinction between active and passive is often less clear (Bauer *et al.*, 2021).

## Is resupination active or passive?

Cardoso *et al*. (2025) explore how and why flower resupination, the 180° inversion, happens in *Phragmipedium vittatum*, a lady’s slipper orchid. The process is very important for pollination in this orchid and is embedded in a complex chain of functional steps: it orientates a specialized landing platform for pollinating hoverflies; the vertical orientation is essential because the flower also produces a slippery landing surface which provokes downward slipping of the flies into the inverted and cramped flask-like labellum. Here the hoverflies cannot hover but are obliged to bump into sticky pollen; they escape with attached pollen to another flower and repeat the process (Cardoso *et al.*, 2022). As if all this was not complex enough, the flower surface is dotted with fake aphids where hoverflies instinctively lay eggs so that emergent larvae can feast on them (Cardoso *et al.*, 2022). You couldn’t make it up! The turning upside down of the labellum is therefore crucial for facilitating this chain of events: luring, landing, slipping, blundering about, transferring pollen, and escaping to repeat the process on another flower (Cardoso *et al.*, 2023).

Previous work has shown that auxins and indole acetic acid (IAA) drive active gravitropic growth of the pedicel downwards (Nair and Arditti, 1991). Active asymmetric growth of raphides in the pedicel produces the required initial curve prior to downward gravitropic extension growth (Dines and Bell, 1994). The gravitropic and raphide growth mechanisms sound like a reasonable explanation for the full mechanism, but Cardoso *et al*. (2025) suspected that the mass of the apical flower and its potential energy contribute importantly to the downwards orientation. This is likely to ensure the reliability of the inversion, in turn ensuring a higher probability of pollination. This is interesting since it would mean that a passive element contributes to the inverting process. We shall see that this has important implications for the robustness of the mechanism and its possible variability. We will also see how viewing complex functions as ‘living machines’ can offer new insights into bio-inspired technologies.

## The details of the resupination process

The authors selected a site in the Cerrado biome of Goiás state in Brazil where a population of the threatened *P. vittatum* lady’s slipper orchid is known to show different degrees of resupination—some hardly invert at all, some invert a bit, and some invert completely. They previously described that most flowers were fully resupinate, with ~10% of flowers being only slightly or somewhat inverted (Cardoso *et al*., 2023).

Cardoso *et al*. (2025) showed that the timing of resupination, the increase in flower mass, and the timing of anthesis were all important for the flowers to open in the correct vertical position. They investigated which parts of the flower had the highest mass and how the removal of individual flower parts would influence resupination. After removing all flower parts, there was no resupination; when only the labellum was removed, there was only a 30% chance of resupination. Removal of any other individual parts affected the chance of resupination considerably less. All this indicates that it was the mass of the labellum (~1 g) that was most strongly linked to the complete 180° resupination of the flower.

Finally, observations of the flower pedicel before resupination and anthesis suggest that active gravitropic growth of the tiny stalk (pedicel) bearing the flower adjusts the angle of the stalk a little downwards. The authors also noted a tendency for a lateral movement of the stalk. These results suggest that active processes position the flower in an initial downwards-pointing position, probably acting as a slight off-vertical bending moment which effectively primes the organization to hinge downwards after an increase in the mass of the flower. The increase in mass of the labellum occurs during the night and early morning before anthesis when water transport hydrates the tissue of the labellum. The slender compliant part of the pedicel deforms, acting as a hinge under bending and probably torsion, and the flower swings around with gravity into the inverted position.

From a practical viewpoint, this study shows how a complex biomechanical process and its inference can be determined without any biomechanical measurements in the field. For example, the hinge-like properties of the tiny flower pedicel would be very difficult to measure *in situ* under field conditions. Instead, sensibly planned observations and a statistical approach offered the chance to gather evidence that a mix of active and passive processes is at play.

## Success and failure

Most flowers observed showed a full 180° movement, meaning that the mechanism is relatively robust if the main ‘aim’ is for a full vertical orientation. It is interesting that this is a passive movement using a simple mass that completes the process. One could guess that the passive counterweight mechanism is a reliable way of making sure that the final orientation is as vertical as possible. Active orientational growth could probably be driven off-course and off-vertical if rain, wind, and other environmental perturbations were to intervene. All these intricate movements take place in the open air, of course, not in the lab! The landing area and slippery surface must be vertical so that hoverflies fall into the chamber below.

The authors dip into Mayr’s essay on functional causality, and the exploration of biological phenomena employing proximate (individual and mechanistic process) versus ultimate (a population and evolutionary explanation) views of describing biological phenomena (Mayr, 1961). Choosing another favourite classic from the evolutionary literature, the developmental mix of active and passive processes in the lady’s slipper may have a bearing on Gould and Vrba’s essay on exaptation (Gould and Vrba, 1982). In my view, this is an underused term, which identifies a structure that takes on a different function during evolution after some modification—the classic example being bird feathers (for flight) as derived from dinosaur feathers (for thermoregulation). I wonder whether the slightly and partially resupinated flowers really represent outright ‘failures’. Seeing the degree and margin of precision in this orchid story (1 g for the labellum and the importance of bending and torsion moments depending on force and distance), it would not take much of a change in mass of the counterweight or angle or length of the pedicel to modify the orientation of the landing place, and possibly the kind of pollinator having another kind of locomotion and life history. In plant biomechanics, especially observations under real-world, wild conditions, we notice that active–passive mechanisms that seem perfect under set-piece conditions in the lab, such as twining and attachment, do not always work ‘perfectly’ in the hurly burly of life in the wild (Lehnebach *et al.*, 2022; Rowe *et al.*, 2023).

For the lady’s slipper example, most individuals invert fully, but others show variations; these variations could be small differences in active development or passive factors such as water availability just prior to anthesis that could modify the mass of the labellum and the overall fine-tuned ensemble of component traits and the extent of inversion. Such mechanistic differences could represent the means by which the lady’s slipper spreads its bets for modifying and escaping a tight functional regime with specific pollinators faced with unpredictable environmental conditions. Linking plant development step-by-step with precise functional mechanisms at this morphological scale rings true with the view that jointly considering development and function might challenge Mayr’s separation of proximate and ultimate processes for understanding cause and effect (Brown, 2020). Furthermore, such approaches might identify and explore current ‘failures’ as future avenues of escape from adaptive valleys via exaptive changes in function.

## Our living machines

The authors refer to their findings as a ‘working machinery of flower orientation’. I like this. Stepping back and viewing plants and their development as machines is an interesting exercise. A ‘living machines’ view is now established for bio-inspired approaches seeking to invent new technologies (Box 1). In areas such as soft robotics and multi-materials science, researchers are increasingly turning to plants (Mazzolai *et al.*, 2014; Mazzolai, 2017).

Box 1. **Plants, living machines, and soft robotics**

Recent soft robots based on climbing plants are now capable of autonomous, tropism-like growth, sensing light and gravity, and even adapting mechanical properties of their artificial stem during growth and twining around supports (Del Dottore *et al.*, 2024). Other new artefacts include artificial tendrils that can grab objects and tighten their hold by electro-osmotic changes (Must *et al.*, 2019) as well as shape-memory polymers within artificial multi-material tendrils that can perform complex movements by smart internal design (Farhan *et al.*, 2023). Further examples include a climbing cactus—to our knowledge the first time that different developmental stages of a plant have been integrated within bioinspired multi-material plant stems. The stems can undergo tropism-like movements and change cross-sectional shape and mechanical properties, without any energy input other than environmental water availability and changes in ambient temperature (Soffiatti and Rowe, 2020; Bastola *et al.*, 2021a, b); possibly the first instance of what we call in our lab proper ‘Robo-Devo!’ Other living machines use bio-inspired micro-spines from climbing plants as adhesion mechanisms in a range of applications (Fiorello *et al.*, 2020). Recent plant-like artefacts can even generate triboelectric energy (Meder *et al.*, 2020). Most of these bio-inspired inventions, like their bioinspired sources, have active and passive elements embedded in their complex overall functioning functions.

Cardoso *et al*. (2025) describe a remarkable combination of traits as a complex living machine using a mix of active versus passive processes. We are all enchanted by the functionality of these exceptional orchid mechanisms and the remarkable integration of traits that make up these living machines. As sessile, autotrophic organisms, they show that plants do smart stuff without having a brain—even outwitting animals that do.

A take-home message is that stepping back and considering complex, integrated biological traits as machines can fuel new interdisciplinary research towards novel technologies. This account reinforces the rich breadth and depth of development and functionality that plants have to offer for novel bio-inspired applications.

Finally, the importance of our living machines in their threatened natural habitats, such as the Cerrado biome of Brazil (Sant’Anna-Santos, 2023), is yet one more potent argument for why natural biodiversity is a wonderful resource, not only for new kinds of ecosystem services, food security, and pharmaceuticals, but for our new generations of otherwise unimaginable bioinspired technologies.
